# Discovery of Biomarkers Related to Interstitial Fibrosis and Tubular Atrophy among Kidney Transplant Recipients by mRNA-Sequencing

**DOI:** 10.3390/jpm13081242

**Published:** 2023-08-10

**Authors:** Hyun Kyung Lee, Na Hyun Jung, Da Eun Lee, Hajeong Lee, Jaeseok Yang, Yon Su Kim, Seung Seok Han, Nayoung Han, In-Wha Kim, Jung Mi Oh

**Affiliations:** 1 College of Pharmacy and Research Institute of Pharmaceutical Sciences, Seoul National University, Seoul 08826, Republic of Korea; hazel529@snu.ac.kr (H.K.L.);; 2 Division of Nephrology, Department of Internal Medicine, Seoul National University Hospital, Seoul 03080, Republic of Koreayonsukim@snu.ac.kr (Y.S.K.);; 3 Internal Medicine, Seoul National University College of Medicine, Seoul 03080, Republic of Korea; 4 Transplantation Center, Seoul National University Hospital, Seoul 03080, Republic of Korea; 5 Division of Nephrology, Department of Internal Medicine, Yonsei University College of Medicine, Seoul 03722, Republic of Korea; 6 College of Pharmacy, Jeju National University, Jeju 63243, Republic of Korea

**Keywords:** interstitial fibrosis and tubular atrophy, mRNA-sequencing, kidney transplantation, peripheral blood mononuclear cell, differentially expressed gene

## Abstract

Interstitial fibrosis and tubular atrophy (IF/TA) after kidney transplantation causes a chronic deterioration of graft function. IF/TA can be diagnosed by means of a graft biopsy, which is a necessity as non-invasive diagnostic methods are unavailable. In this study, we identified IF/TA-related differentially expressed genes (DEGs) through next-generation sequencing using peripheral blood mononuclear cells. Blood samples from kidney transplant recipients undergoing standard immunosuppressive therapy (tacrolimus/mycophenolate mofetil or mycophenolate sodium/steroid) and diagnosed as IF/TA (*n* = 41) or normal (controls; *n* = 41) at their one-year protocol biopsy were recruited between January of 2020 and August of 2020. DEGs were derived through mRNA sequencing and validated by means of a quantitative real-time polymerase chain reaction. We identified 34 DEGs related to IF/TA. *ADAMTS2*, *PLIN5*, *CLDN9*, and *KCNJ15* demonstrated a log_2_(fold change) of >1.5 and an area under the receiver operating characteristic curve (AUC) value of >0.6, with *ADAMTS2* showing the largest AUC value and expression levels, which were 3.5-fold higher in the IF/TA group relative to that observed in the control group. We identified and validated DEGs related to IF/TA progression at one-year post-transplantation. Specifically, we identified *ADAMTS2* as a potential IF/TA biomarker.

## 1. Introduction

Organ failure is an emerging problem with the aging of society, and organ transplantation represents the optimal treatment for end-stage organ failure. Although technological developments and the use of immunosuppressive agents have decreased the incidence of acute rejection, chronic allograft nephropathy has become the primary cause of graft dysfunctions. Interstitial fibrosis and tubular atrophy (IF/TA) are collectively defined as a disease without a specific etiology [1] and are present in approximately 30% of patients one year after and in almost all patients ten years after kidney transplantation. In some patients, IF/TA develops early, possibly contributing to early graft dysfunction and serving as an indicator by which to predict allograft failure [2]. IF/TA is diagnosed through an invasive kidney biopsy after kidney transplantation. Although the biopsy performed in stable patients causes a low incidence of serious complications [3], there remains a risk of bleeding, and the process causes discomfort. Diagnosis through a biopsy also complicates an early diagnosis of IF/TA. Moreover, the mechanisms associated with IF/TA pathology have not yet been determined, resulting in a lack of effective diagnosis and treatment strategies. Therefore, identifying changes in the gene expressions associated with IF/TA onset and progression could provide a better understanding of the complex status related to IF/TA in kidney transplantation.

Altered levels of transcribed mRNA represent candidate biomarkers stemming from their ability to signal epigenetic changes based on environmental alterations, such as disease progression [4]. Numerous studies have attempted to identify mRNA biomarkers related to IF/TA after kidney transplantation.

Most previous studies have primarily focused on gene expression analysis with allograft tissue [5,6,7,8,9,10,11,12,13,14,15,16,17]. However, applying these findings to blood samples is challenging due to significant differences in gene expression profiles between biopsy tissue and blood samples [17,18,19]. Therefore, specific research targeting blood samples is necessary to identify blood biomarkers. While some gene expression studies have used blood samples [17,19,20], discrepancies in differentially expressed genes (DEGs) have been observed, and no previous study has performed whole mRNA sequencing on blood samples.

For instance, Mas et al. [17] collected peripheral blood mononuclear cells (PBMCs) from a chronic allograft nephropathy (CAN) group (*n* = 11) and a stable renal function group (*n* = 20). They used a quantitative real-time reverse transcription polymerase chain reaction (qRT-PCR) assessment to assess the expression levels of transforming growth factor β (*TGF-β*), the epidermal growth factor receptor (*EGFR*), and angiotensinogen (*AGT*). However, no significant differences were found in the expression levels of these genes in PBMC samples despite significant differences being observed using microarray analysis of allograft tissues. Similarly, Kurian et al. [19] collected PBMCs from kidney transplant recipients (*n* = 77) with different IF/TA grades (0, 1, 2, and 3). They used microarray analysis and identified 393 DEGs related to mild IF/TA and 63 DEGs related to moderate/severe IF/TA. The top network related to IF/TA was found to be associated with the immune response. Interestingly, their DEG profiles in blood samples were different from those in their earlier study [16] using allograft tissue samples. In another study, Matz et al. [20] collected whole blood samples from an IF/TA group (*n* = 33) and a stable graft function group (*n* = 40). They conducted RT-PCR analysis and found that chemokine interleukin (IL)-8 was downregulated in IF/TA patients.

Although there is strong evidence of an association between altered gene expression and IF/TA progression, their gene expression results relating to IF/TA were inconsistent. Furthermore, because these studies mainly analyzed gene expression outcomes in biopsy samples, their clinical applications are limited. On the other hand, PBMCs are widely used in both research and clinical applications. Additionally, there has been no next-generation sequencing (NGS) research analyzing the mRNA expression related to IF/TA. Unlike a microarray or a qPCR analysis, which tends to focus on specific genes, NGS allows for a broad survey of the gene-expression landscape. Therefore, in this study, we investigated possible IF/TA-related DEGs through the mRNA-sequencing of PBMCs derived from patients with and without IF/TA one year after their kidney transplant.

## 2. Materials and Methods

### 2.1. Study Population

We included 82 patients (aged: 18–70 years) who underwent a one-year protocol biopsy after receiving a kidney transplant at a tertiary teaching hospital in South Korea from 2015 to 2018. Exclusion criteria included patients who were enrolled in other clinical trials, including non-Korean individuals aged < 18 or >70, recipients of non-first kidney transplants, those with follow-up loss, patients with active infections during the one-year protocol biopsy, and patients diagnosed with malignant tumors after kidney transplantation. Additionally, patients with biopsy results differing from the one-year biopsy were excluded if additional biopsies were performed. Each diagnosis for allograft pathology was conducted according to the Banff classification [21]. Patients diagnosed as IF/TA only or IF/TA with borderline changes (suspicious acute T cell-mediated rejection) were included in the IF/TA group. Patients without any allograft rejections, including IF/TA or disease recurrence, were included in the control group. Demographic characteristics (age at transplant date, sex, body mass index [BMI] at transplant date, dialysis information, original disease, and postoperative days), kidney transplantation information (donor type and age), medication information, and biopsy information were collected. The estimated glomerular filtration rate (eGFR) using the CKD-EPI equation on the one-year protocol biopsy date and blood sampling date were collected to assess changes in kidney status between the two time points. This study was approved by the Institutional Review Board of Seoul National University Hospital (IRB No. 1904-094-1027) and conducted according to the recommendations of the Declaration of Helsinki. Informed consent was obtained from all participants.

### 2.2. Sample Preparation

The blood samples were collected between January 2020 and August 2020. Peripheral blood (10 mL) was collected in an EDTA tube (BD, Franklin Lakes, NJ, USA) during an outpatient visit and stored in a refrigerator. During transport, it was placed in an icebox to preserve the mRNA against heat degradation. Mononuclear cells were isolated from peripheral blood within 3 h of collection. A lymphocyte–separation medium (3 mL; MP Biomedicals, Solon, OH, USA) stored at room temperature was added to a 15-mL centrifuge tube, followed by the addition of 2 mL of phosphate-buffered saline (PBS) (Bioworld, Dublin, OH, USA) and 2 mL of blood. The tube was then centrifuged at 400× *g* for 30 min, after which the top layer was removed by aspiration, and the mononuclear cells were then transferred to a new 15-mL centrifuge tube. After adding 10 mL of PBS, a second tube was centrifuged at 400× *g* for 10 min, followed by an aspiration to remove the liquid phase. These steps were performed twice simultaneously in order to generate two pellets that were subsequently combined. The RNAlater reagent (150 µL; QIAGEN, Germantown, MD, USA) was then added to the pellet, which was stored at −70 °C. RNA was extracted from the mononuclear cells using a RiboPure-blood kit (RiboPure RNA Purification Kit, Blood, Thermo Fisher Scientific, Waltham, MA, USA). DNase (Invitrogen, Vilnius, Lithuania) was used to remove genomic DNA (gDNA).

### 2.3. mRNA-Sequencing

RNA purity was analyzed using a NanoDrop 8000 spectrophotometer (Thermo Fisher Scientific), and the total RNA integrity was determined according to the RNA integrity number (RIN) using a 2100 Bioanalyzer instrument (Agilent Technologies, Santa Clara, CA, USA). A RIN exceeding 7 and a 28S/18S rRNA ratio exceeding 1.5 were set as acceptable levels. Successful gDNA removal was verified using a Bioanalyzer. mRNA-sequencing libraries were prepared using the Truseq stranded mRNA library prep kit (Illumina, San Diego, CA, USA) according to the manufacturer’s instructions. mRNA was purified and fragmented from total RNA (1 μg) using Oligo(dT) magnetic beads, which were then primed and cleaved using random hexamers. RNA fragments were reverse-transcribed into first-strand cDNA using reverse transcriptase, with a single ‘A’ nucleotide added to the cDNA fragments, followed by an adaptor ligation. The fragments were amplified by PCR, and the quality of the cDNA libraries was verified by capillary electrophoresis (2100 Bioanalyzer, Agilent Technologies). Clustering was performed in a flow cell using a cBot automated cluster generation system (Illumina), after which sequencing was performed at a 2 × 100-bp read length using a Novaseq 6000 sequencing system (Illumina). mRNA-sequencing was performed at a DNA Link (Seoul, Republic of Korea). mRNA-sequencing data were submitted to NCBI’s Gene Expression Omnibus database (GSE218048).

### 2.4. DEG Analysis

Reads were mapped to the reference genome using Tophat (v2.0.13, RRID:SCR_013035) [22], and DEGs were derived from aligned results using Cuffdiff (v2.2.1, RRID:SCR_001647) [23,24]. A geometric method was used for library normalization, and a pooled method was utilized for dispersion estimation. Genes that only contained enough reads with a raw read count of 10 or higher levels were extracted. Genes satisfying the condition of |log_2_(fold change)| ≥ 1 were selected. The cut-off was set at a false discovery rate (FDR)-adjusted *p*-value (*q*-value) of <0.1. For all the genes, log_2_(fold change) and log_10_(*q*-value) are drawn in a volcano plot.

### 2.5. Ontology Analysis

For ontology analysis, genes satisfying the condition |log_2_(fold change)| ≥ 1 were subjected to a Database for Annotation, Visualization, and Integrated Discovery (DAVID) (RRID:SCR_001881) [25] analysis, with “disease”, “gene ontology”, and “pathway” categories selected [26]. The cut-off for the Expression Analysis Systematic Explorer (EASE) score [27] was set to 1, and functional annotations for each category were generated.

### 2.6. Protein–Protein Interaction (PPI) Network Analysis

The PPI network analysis for significant DEGs was conducted using the STRING database (https://string-db.org/) (accessed on 11 July 2023) [28]. The minimum required interaction score was set as 0.4.

### 2.7. qPCR Validation of the Gene-Expression Profiles

To validate the mRNA-sequencing results, qPCR was performed on interested genes. The primer/TaqMan probe combinations for each target sequence were purchased from Thermo Fisher Scientific (Table S1). cDNA was produced using a Superscript II RT-PCR system (Invitrogen, Karlsruhe, Germany) according to the manufacturer’s instructions. The comparative C_t_ method was used to calculate the relative gene expression levels with glyceraldehyde 3-phosphate dehydrogenase (*GAPDH*) used as endogenous control [29]. Relative gene expression levels were determined using the 2^−ΔΔCt^ method.

### 2.8. Statistical Analysis

Patient demographics were presented as the frequency (%), mean ± standard deviation (SD), or median (range). For continuous variables, a *t*-test or the Wilcoxon rank sum test was performed depending on the degree of normality. For non-continuous variables, a chi-squared test or Fisher’s exact test was used. A paired *t*-test was used to assess changes in eGFR between the biopsy and blood sampling dates. In the case of multiple group comparisons, an analysis of variance (ANOVA) or the Kruskal–Wallis test with Dunn’s multiple comparisons was performed.

To evaluate the discriminatory power of DEGs as biomarkers, we calculated the area under the receiver operating characteristic curve (AUC) values. R (v4.0.3) was used for the statistical analyses, and *p* < 0.05 was considered statistically significant.

The association between the expression levels of the most important DEGs and patient clinicopathological factors (age, BMI, eGFR at one-year protocol biopsy, eGFR at blood sampling, sex, dialysis type, original disease, and donor type) was analyzed using a multiple linear regression model with backward elimination. Additionally, the relationship between IF/TA and the expression of the most important DEGs, along with patient clinicopathological factors, was examined using multiple logistic regression with backward elimination.

To enhance the predictability of DEGs for IF/TA, AUC was analyzed for panels of DEGs that exhibited a log_2_(fold change) > 1.5 and AUC values > 0.6. Moreover, the AUC for the model, including DEGs and clinicopathological factors, was calculated. Furthermore, a subgroup analysis using qPCR was conducted by dividing the patients into two groups: an early blood collection group consisting of those who were within two years of their transplant and a late blood collection group consisting of those more than two years after their transplant.

## 3. Results

### 3.1. Baseline Clinical Characteristics of Patients

**Figure 1:**
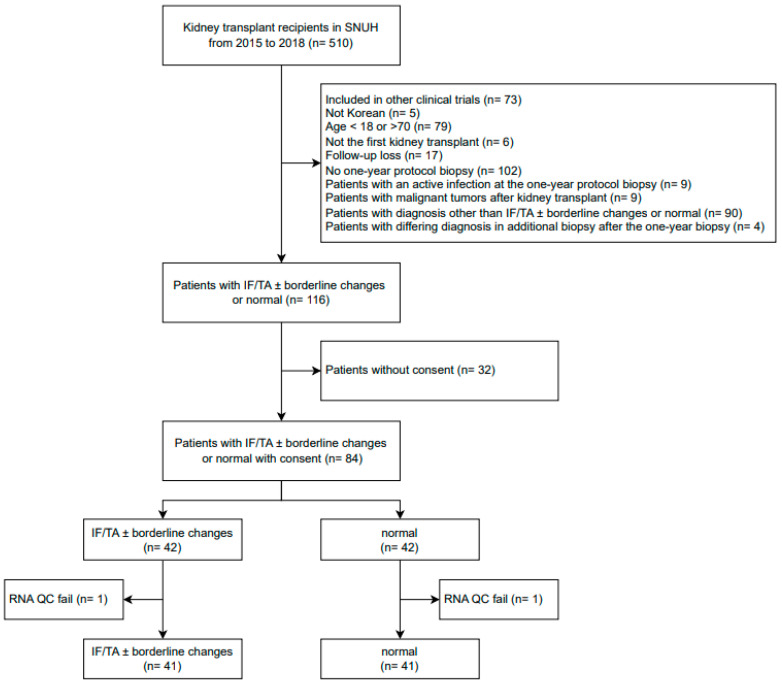
Flow chart of patient enrollment. SNUH, Seoul national university hospital; IF/TA, interstitial fibrosis and tubular atrophy; QC, quality control.

Figure 1 shows the flow chart of patient enrollment. Table 1 shows the baseline characteristics of the IF/TA group (*n* = 41) and the control group (*n* = 41). The clinicopathological features for IF/TA patients and controls are given in Table S2. The median (range) ages of the IF/TA and control groups were 50 (27–67) years and 52 (20–68) years, respectively (*p* = 0.94), with the male proportions of each group at 51.2% and 61.0%, respectively (*p* = 0.50). The proportion of deceased donors was higher in the IF/TA group, although the donor type did not differ between the groups (*p* = 0.06). At the time of transplantation, all patients were on triple therapy with tacrolimus, mycophenolate mofetil, or mycophenolate sodium, and a steroid. Thirty-seven (90.2%) in the IF/TA group and 35 (85.4%) in the control group were maintaining triple therapy upon their one-year protocol biopsy. The mean of the differences in eGFR between the date of biopsy and blood collection was 0.09 (*p* = 0.92), indicating that kidney status did not change significantly between these two time points. The median time between biopsy and sampling was 597 (52–1597) days. Additionally, the distributions of BMI, dialysis type, original disease, donor type, donor age, and postoperative days did not differ between the groups.

### 3.2. DEGs Related to IF/TA

**Figure 2:**
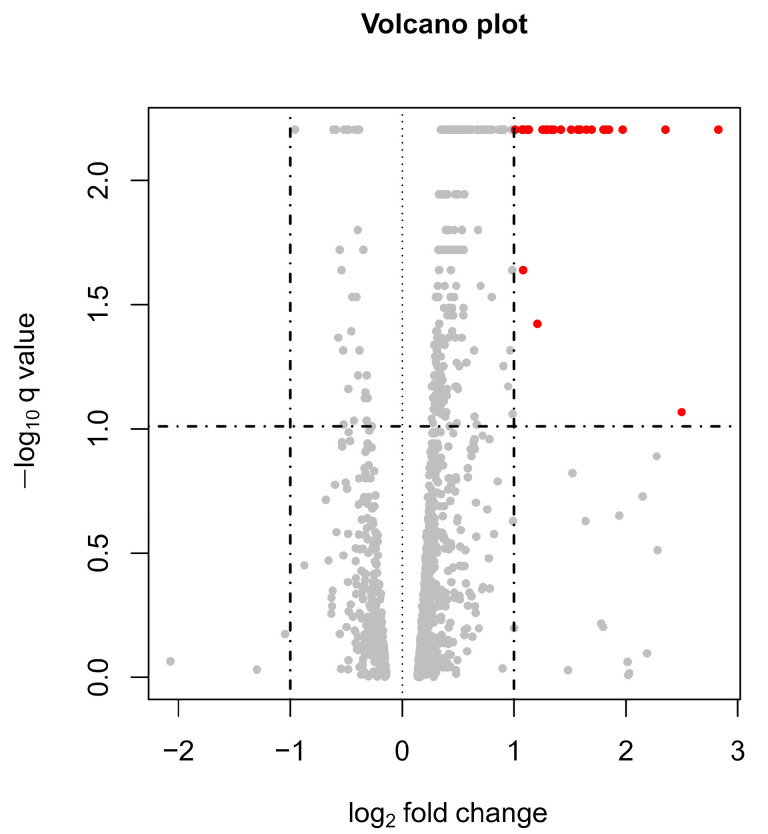
Volcano plot of gene expression levels in PBMCs from IF/TA patients (*n* = 41) and controls (*n* = 41) at their one-year protocol biopsy after kidney transplant. The X-axis represents the fold change in the mRNA expression level in the IF/TA group relative to the control group, and the Y-axis represents the *q*-value (FDR-adjusted *p*-value). Each point represents a gene. The dotted line indicates the cut-off value for a determination as a DEG (|fold change| > 2 and *q*-value < 0.1). Red dots indicate genes that satisfy the DEG criteria.

**Figure 3:**
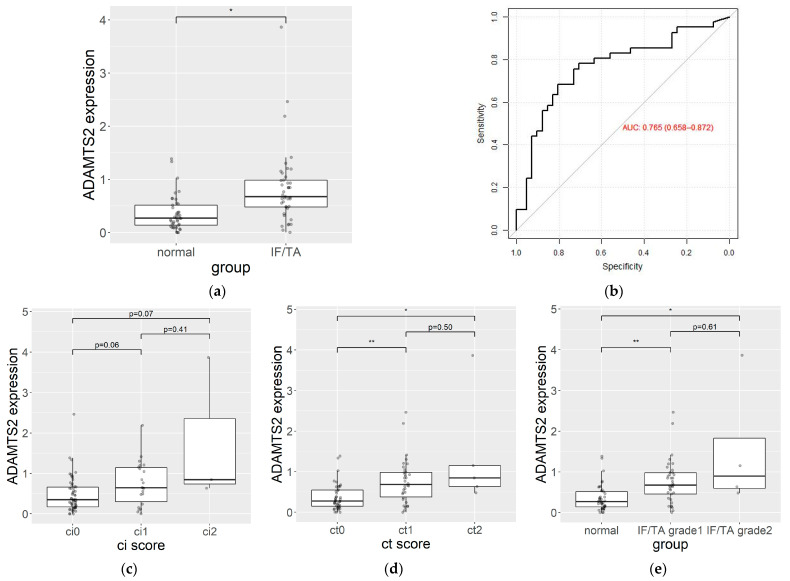
(**a**) *ADAMTS2* expression levels in PBMCs from IF/TA patients (*n* = 41) and controls (*n* = 41) at their one-year protocol biopsy after kidney transplant. Data represent the median and interquartile ranges of the log_2_(FPKM + 1). Dots represent individual data points. (**b**) ROC curve evaluating the sensitivity and specificity of *ADAMTS2* as a biomarker of IF/TA. (**c**) *ADAMTS2* expression level from ci0 (*n* = 57), ci1 (*n* = 22) and ci2 (*n* = 3). (**d**) *ADAMTS2* expression level from ct0 (*n* = 43), ct1 (*n* = 34) and ct2 (*n* = 5). (**e**) *ADAMTS2* expression level from normal (*n* = 41), IF/TA grade 1 (*n* = 37), and IF/TA grade 2 (*n* = 4). AUC, the area under the receiver operating characteristic curve; FPKM, fragments of transcript per kilobase per million reads; * *p* < 0.05, ** *p* < 0.001.

The mean of the total reads count was 63,480,861 (39,039,570–88,876,806), and the mean of the mapping rate to the reference genome was 94.92%. A density plot and a box plot of the FPKM distribution are shown in Figure S1, which indicates there were no batch effects or outlier samples. Figure 2 shows a volcano plot of the gene expression levels. The expression values of 34 genes differed by more than two-fold, satisfying the *q*-value threshold (<0.1). All DEGs showed a higher expression level in the IF/TA group relative to that in the control group (Table 2, Figure 3). Among the 34 DEGs, disintegrin and metalloproteinase with thrombospondin motifs 2 (*ADAMTS2*) showed the highest AUC value (AUC = 0.77; 95% confidence interval: 0.66–0.87), and the average *ADAMTS2* expression was 3.5-fold higher in the IF/TA group compared to the control group. Figure S2 shows the ROC curves of all DEG which showed a log_2_(fold change) of >1.5 and AUC values of >0.6. In the further analysis of *ADAMTS2* expression, when dividing patients by their ci and ct scores and IF/TA grades, we observed that *ADAMTS2* expression levels increased as the score increased. However, these differences were not statistically significant (Figure 3c–e).

To examine the effects of patient clinicopathological factors on *ADAMTS2* expression, a multiple regression analysis was conducted. After backward elimination, only recipient age showed a significant correlation with *ADAMTS2* expression (coefficient = 0.02; 95% confidence interval: 0.005–0.025; *p* < 0.001).

To enhance the predictability of DEGs for IF/TA, we analyzed the AUC for panels of DEGs that exhibited a log_2_(fold change) > 1.5 and AUC values > 0.6. Additionally, a logistic regression with backward elimination was performed to adjust for clinicopathological factors. Consequently, recipient age was associated with IF/TA. We observed a slight improvement in the diagnostic performance of the *ADAMTS2* and claudin 9 (*CLDN9*) panel, as well as the combined *ADAMTS2* model with clinicopathological factors (Table 3, Figure S3).

### 3.3. Ontology Analysis

**Figure 4:**
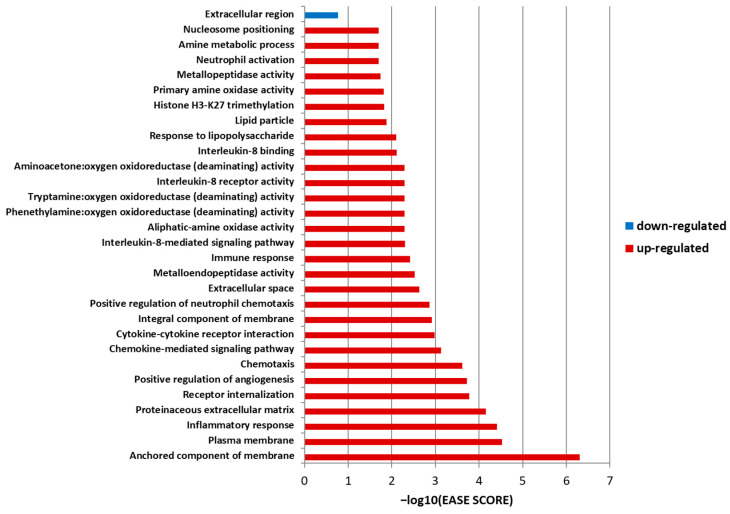
Gene ontology results for IF/TA patients (*n* = 41) and controls (*n* = 41) at their one-year protocol biopsy after kidney transplantation. The X-axis represents the −log_10_(EASE score), and the Y-axis represents the annotation term. The Expression Analysis Systematic Explorer (EASE) score is a modified Fisher exact test in a gene ontology enrichment analysis. A blue bar indicates a term related to the downregulated genes, and red bars indicate the terms related to the upregulated genes with an EASE score of <0.02.

Figure 4 shows the results of the ontology analysis. An analysis of downregulated genes revealed only one term (extracellular region), whereas an analysis of upregulated genes identified 128 terms. The term with the lowest EASE scores was termed an “anchored component of membrane”, and among the top 10 enriched ontology terms, *ADAMTS2* was related to the “proteinaceous–extracellular matrix”. This pathway included alkaline phosphatase, biomineralization-associated (*ALPL*), matrix metalloproteinase 9 (*MMP9*), chitinase-3-like protein 1 (*CHI3L1*), and fibronectin 1 (*FN1*) among DEGs.

### 3.4. PPI Network Analysis

**Figure 5:**
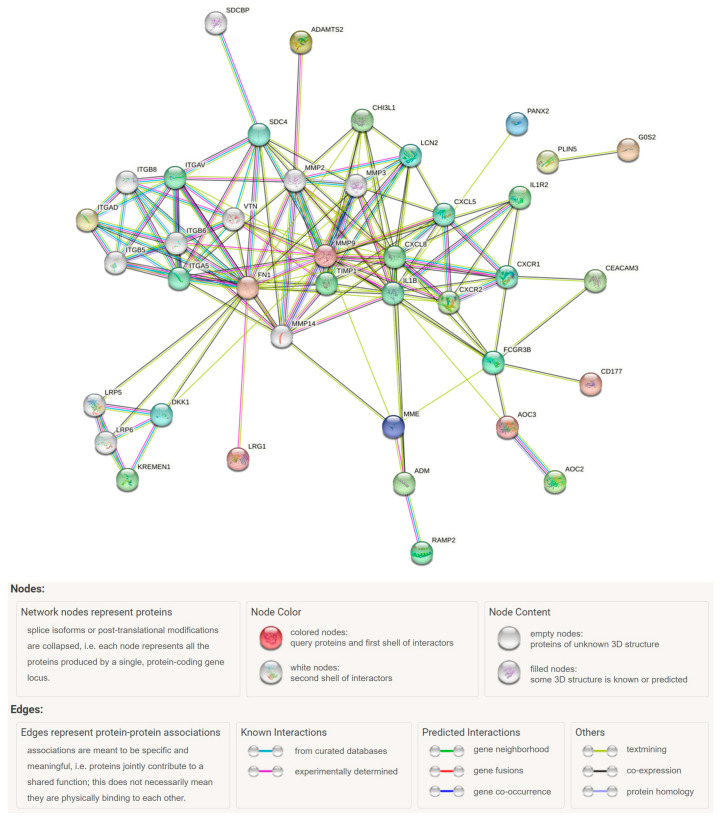
PPI network analysis of IF/TA-associated DEGs (*n* = 34) using the STRING tool.

The network included 52 nodes with 147 interactions, which was significantly more interactions than expected (PPI enrichment *p*-value: < 1.0 × 10^−16^) (Figure 5). Some proteins had notably more interactions than others, representing key nodes. In the network, we identified *FN1*, C–X–C motif chemokine ligand 8 (*CXCL8*), *CHI3L1*, C-X-C motif chemokine receptor 2 (*CXCR2*), adrenomedullin (*ADM*), *ITGAD*, *MMP9*, and Fc gamma receptor IIIb (*FCGR3B*) as key nodes among the DEGs.

### 3.5. qPCR Analysis

The qPCR results for the interested genes, in this case, *ADAMTS2*, *CHI3L1*, *CXCR2*, interleukin 1 receptor type 2 (*IL1R2*), leucine-rich alpha-2-glycoprotein 1 (*LRG1*), and *MMP9*, showed good agreement with the mRNA-sequencing results. All genes were upregulated in the IF/TA group relative to the control group, and *ADAMTS2* showed the highest fold change (2.20; *p* < 0.001) (Table S3).

The fold change in *ADAMTS2* in the subgroup analysis performed for patients with an early blood collection within 2 years after transplantation (IF/TA [n = 15]; control [*n* = 10]) was 1.3 (*p* = 0.13), and for patients with late blood collection, more than 2 years after transplant (IF/TA [*n* = 26]; control [*n* = 31]) was 2.9 (*p* < 0.001).

## 4. Discussion

In this study, we identified 34 DEGs related to IF/TA after kidney transplantation using NGS. To the best of our knowledge, this is our first study to identify and evaluate IF/TA-related DEGs following kidney transplantation using mRNA-sequencing in PBMCs.

Previous studies investigating gene expression in relation to IF/TA in blood samples primarily utilized RT-PCR [17,20] or a microarray analysis [19] to examine specific genes, resulting in variations in the DEGs identified across these studies. Additionally, it was noted that DEGs identified in blood samples differed from those found in biopsy samples [17,19], highlighting the need for whole mRNA sequencing studies specific to blood samples.

In line with a previous study focusing on blood samples [20], *IL-8* (*CXCL8*) was also found to be associated with IF/TA in our study. However, its direction for the regulation of *CXCL8* differed between the previous study and our findings. While previous research showed the downregulation of *CXCL8* in IF/TA, our study revealed the upregulation of *CXCL8* in IF/TA. Matz et al. proposed that IL-8-producing cells could migrate to allograft and secreted chemokines, leading to lower expression levels in the blood. This inconsistency in the literature suggests a potential association between IF/TA and *CXCL8*, with its expression in blood possibly varying over time. Future research comparing expression levels at various time points after kidney transplantation in both blood and allograft tissue might be valuable. In another study using microarray analysis of allograft tissues [17], genes related to fibrosis and extracellular matrix deposition, including *MMP9*, were found to be up-regulated, but blood samples did not show a significant difference. However, our study demonstrated the significant up-regulation of *MMP9* on PBMC. This strengthens the evidence supporting the association of *MMP9* with IF/TA despite the relatively small differences in expression levels in the blood samples. Additionally, a previous study using microarray analysis of PBMC [19] identified a larger number of DEGs compared to our study, and these DEGs were different between the studies. However, the immune response pathway was consistently identified as one of the key pathways in both the previous study and our study.

Among the 34 DEGs, including *ADAMTS2*, perilipin 5 (*PLIN5*), *CLDN9*, and the potassium inwardly-rectifying channel, subfamily J, member 15 (*KCNJ15*) showed log_2_(fold change) values of >1.5 and an AUC value of >0.6, with *ADAMTS2,* demonstrating the largest AUC and suggesting its potential utility as a biomarker for IF/TA. We observed an increased tendency of the *ADAMTS2* expression with IF/TA progression, although these differences were not statistically significant due to the small sample size with severe IF/TA. The AUC of *ADAMTS2* in this study (0.765) might not be sufficient for a standalone diagnosis. While its standalone diagnostic utility might be limited, *ADAMTS2* could potentially contribute to the development of a comprehensive diagnostic panel for the IF/TA. Currently, there is no widely used blood-based biomarker in the clinic for a diagnosis of IF/TA, as its diagnosis primarily relies on histopathology obtained through a biopsy. Based on these considerations, it is reasonable to suggest that the *ADAMTS2* expression in PBMC has a clinical value as a biomarker candidate. An additional analysis using a DEG panel and an adjusted model was conducted to enhance the diagnostic performance of DEGs, but only a marginal improvement was observed. These findings suggest that *ADAMTS2* was the most significant biomarker candidate for diagnosing IF/TA progression in this study.

ADAMTS2 is a procollagen N-endopeptidase that cleaves amino-propeptides and induces the formation of collagen fibrils. ADAMTS2 is mainly secreted from fibroblasts and plays important roles in fibrosis, with elevated expression levels observed in fibrotic lesions [30] and diseases such as cardiac hypertrophy [31], osteoarthritis [32], and squamous cell carcinoma [33]. Additionally, a previous study showed that elevated *ADAMTS2* expression levels in biopsy samples at three months after kidney transplantation preceded a diagnosis of IF/TA at six months [11]. These findings and those of the present study suggest that an increased *ADAMTS2* expression in PBMCs could represent a molecular marker of IF/TA in the transplanted kidney. PLIN5, a lipid droplet that targets proteins, is highly expressed in oxidative tissues [34] and exerts a protective effect against oxidative stress upon the upregulation of its expression [35]. Because oxidative stress is related to IF/TA progression in allograft tissue [36,37], the upregulation of *PLIN5* could represent a defense mechanism against IF/TA-related oxidative stress.

*CLDN9* and *KCNJ15* encode proteins that are integral components of the plasma membrane. CLDN9 is a member of the claudin family and is a component in renal tight junctions [38]. Although a relationship between CLDN9 and fibrosis has not yet been determined, in the present study, we found that *CLDN9* expression was elevated in samples from the IF/TA group, suggesting its possible involvement in IF/TA progression after transplantation. KCNJ15 is expressed in cells of various organs, including the kidney, and is involved in regulating the acid–base balance of the kidney [39]. Additionally, *KCNJ15* overexpression is associated with type 2 diabetes [40]. Given the role of acidosis and diabetes in renal fibrosis [41,42], it is possible that *KCNJ15* is related to IF/TA progression according to its role in these areas.

Among the other genes showing either a log_2_(fold change) of >1.5 or an AUC > 0.6, *MMP9* and *FN1* encode proteins that are associated with the extracellular matrix [43,44], and play an important role in renal interstitial fibrosis [45]. MMP9 is a metalloenzyme that breaks down collagen, and FN1 is a glycoprotein involved in extracellular matrix formation. Previous studies have identified *MMP9* as a DEG in IF/TA patients [12,17], and *Fn1* was identified as a DEG in a study employing an animal model of renal atrophy or allograft damage [46]. The remaining genes in this category have been reported to be associated with inflammatory or immune response pathways [47,48,49,50,51], which are important pathways linked to IF/TA progression [10]. Amine oxidase copper-containing 3 (*AOC3*), previously identified as an IF/TA-related DEG [12,17], and carcinoembryonic antigen-related cell adhesion molecule 3 (*CEACAM3*) expression were reportedly upregulated in PBMCs from idiopathic pulmonary fibrosis patients [52].

Other DEGs that were significantly upregulated in IF/TA patients but showed a log_2_(fold change) of <1.5 and an AUC of <0.6 included several genes associated with renal or fibrotic diseases. Specifically, previous studies identified *CXCL8* as a DEG in IF/TA patients [14,20] and *Chi3l1* was upregulated in a mouse model of renal atrophy [53]. Additionally, previous studies have reported *ADM* upregulation in human proximal tubular epithelial cells cultured under hypoxic conditions [54], including maltase–glucoamylase (*MGAM*) upregulation in idiopathic interstitial pneumonia patients [55] and vanin 3 (*VNN3*) upregulation in clear-cell renal cell carcinoma patients [56]. Collectively, these findings support an association between the DEGs identified in the present study and IF/TA. Although some genes have not been identified as being directly related to IF/TA, they reportedly play roles in fibrosis, inflammation, and/or renal diseases, suggesting their potential value as IF/TA biomarkers.

We conducted a PPI network analysis using the SPRING database and identified significant interactions among the DEGs. The network based on DEGs revealed the presence of multiple MMP gene families, and *ADAMTS2* was found to have interactions with these genes. The most significantly identified gene in our study was *ADAMTS2*, which was found to be associated with the ontology term “extracellular matrix”, which emerged as an important term in the analysis. Previous studies have also consistently reported an association between genes related to the extracellular matrix and IF/TA [10,11,12,14,17]. This suggests that genes associated with the extracellular matrix have the potential to be biomarkers for IF/TA progression and candidates for further studies as potential drug targets.

This study has several limitations. First, the timing of the biopsy and blood sampling differed, ranging from 52 to 1597 days, meaning that subclinical fibrosis could be present after the biopsy. This limitation is inevitable in a retrospective case–control study. However, because IF/TA progression is irreversible [12], this likely did not significantly affect the study results. In order to address this limitation, a subgroup analysis was conducted by dividing the patients into an early blood collection group consisting of those who were within two years of their transplant and a late blood collection group consisting of those more than two years after their transplant. Although there was no significant difference in the *ADAMTS2* expression level in the early blood collection group, the consistent direction of the fold changes between the two subgroup analyses could help compensate for the limitations arising from the different sampling timing. A further prospective study with a long study period during which blood samples were collected immediately after the one-year protocol biopsy should be conducted to collect samples with similar characteristics. Second, causal relationships between gene expression and IF/TA onset/progression could not be established in this study. Gene expression can be altered as a compensatory mechanism in relation to the occurrence of IF/TA. This is a common problem in retrospective gene expression studies; therefore, these considerations should be taken into account when interpreting the results of this study. When using DEGs as biomarkers, only associations between gene expression and IF/TA progression are required. However, care should be taken when using DEGs as therapeutic targets, as some DEGs might exert protective effects against IF/TA. Therefore, further studies that analyze gene expression levels at multiple time points, including expression data prior to the biopsy, should be undertaken in order to more precisely elucidate the mechanisms associated with IF/TA progression. Third, no external validation was performed in this study; accordingly, further evaluations are needed to replicate these results.

This study has the following strength. Unlike previous studies [5,6,7,8,9,10,11,12,17,19,20] that have evaluated gene-expression profiles mainly via biopsy samples with microarrays, the present study used PBMCs, which are more feasible for use in clinical settings. Furthermore, the present study analyzed the expression levels of all genes using NGS, allowing a broader evaluation of the overall gene-expression profiles in the IF/TA and control groups [57].

## 5. Conclusions

In summary, this study identified 34 IF/TA-related DEGs in PBMCs. Among them, *ADAMTS2* demonstrated the highest differential gene expression level between IF/TA patients and the controls, suggesting that it can serve as a potential biomarker for IF/TA diagnosis.

## Figures and Tables

**Table 1:** Baseline characteristics of IF/TA patients (*n* = 41) and controls (*n* = 41) at their one-year protocol biopsy after kidney transplant.

	IF/TA (*n* = 41)	Control (*n* = 41)	*p*
Age (y) at transplant date, median (range)	50 (27–67)	52 (20–68)	0.94
Male, *n* (%)	21 (51.2)	25 (61.0)	0.50
BMI (kg/m^2^, mean ± SD) at transplant date	23.3 ± 4.0	23.4 ± 3.2	0.84
Dialysis type, *n* (%)			0.60
No dialysis	9 (22.0)	10 (24.4)	
Hemodialysis	27 (65.9)	29 (70.7)	
Peritoneal dialysis	5 (12.2)	2 (4.9)	
Original disease, *n* (%)			0.75
Diabetes	8 (19.5)	9 (22.0)	
Glomerular nephritis	13 (31.7)	14 (34.1)	
Hypertension	2 (4.9)	2 (4.9)	
Polycystic kidney disease	7 (17.1)	3 (7.3)	
Others	3 (7.3)	6 (14.6)	
Unknown	8 (19.5)	7 (17.1)	
Donor type, *n* (%)			0.06
Deceased	18 (43.9)	8 (19.5)	
Living/Related	15 (36.6)	22 (53.7)	
Living/Unrelated	8 (19.5)	11 (26.8)	
Donor age (y, mean ± SD)	49.8 ± 12.3	46.1 ± 13.3	0.20
Missing, *n* (%)	3 (7.3)	0 (0)	
Postoperative days, median (range)	814 (428–1951)	1103 (508–2008)	0.14
Immunosuppressants at protocol biopsy, *n* (%)			0.48
Tac/MMF or MPS/steroid	37 (90.2)	35 (85.4)	
Tac/mizoribine/steroid	3 (7.3)	6 (14.6)	
Tac/steroid	1 (2.4)	0 (0)	
IF/TA grade, *n* (%)		N/A	N/A
Grade 1	37 (90.2)		
Grade 2	4 (9.8)		
Banff score ci, *n* (%)			< 0.001
0	17 (41.5)	40 (97.6)	
1	21 (51.2)	1 (2.4)	
2	3 (7.3)	0 (0)	
Banff score ct, *n* (%)			< 0.001
0	2 (4.9)	41 (100.0)	
1	34 (82.9)	0 (0)	
2	5 (12.2)	0 (0)	
Previous biopsy-proven acute rejection, *n* (%)	3 (7.3)	1 (2.4)	0.61
Delayed graft function, *n* (%)	2 (4.9)	0 (0)	0.47
Renal artery stenosis, *n* (%)	1 (2.4)	0 (0)	1.0
eGFR (mL/min/1.73 m^2^, mean ± SD)			
On one-year protocol biopsy day	61.8 ± 13.3	65.7 ± 11.7	0.17
On blood collection day	62.4 ± 13.5	65.26 ± 10.8	0.30

BMI, body mass index; ci0, interstitial fibrosis in up to 5% of the cortical area; ci1, interstitial fibrosis in 6 to 25% of the cortical area (mild interstitial fibrosis); ci2, interstitial fibrosis in 26 to 50% of the cortical area (moderate interstitial fibrosis); ct0, no tubular atrophy; ct1, tubular atrophy involving up to 25% of the area of cortical tubules; ct2, tubular atrophy involving 26 to 50% of the area of cortical tubules; eGFR, estimated glomerular filtration rate; IF/TA, interstitial fibrosis and tubular atrophy; MMF, mycophenolate mofetil; MPS, mycophenolate sodium; Tac, tacrolimus; SD, standard deviation; N/A, not available.

**Table 2:** DEGs identified in IF/TA patients relative to controls at one-year protocol biopsy after kidney transplantation.

Gene ID	AUC (95% CI)	log_2_( Fold Change)	Absolute Fold Change	*p*	*q*
*ADAMTS2*	0.77 (0.66–0.87)	1.82	3.52	0.00005	0.00625
*FN1*	0.69 (0.58–0.81)	1.08	2.12	0.00005	0.00625
*PLIN5*	0.67 (0.55–0.79)	1.84	3.57	0.00005	0.00625
*CLDN9*	0.66 (0.55–0.78)	1.57	2.97	0.00005	0.00625
*IL1R2*	0.66 (0.54–0.78)	1.42	2.68	0.00005	0.00625
*KREMEN1*	0.64 (0.52–0.77)	1.29	2.44	0.00005	0.00625
*LRG1*	0.64 (0.52–0.76)	1.13	2.19	0.00005	0.00625
*KCNJ15*	0.63 (0.51–0.76)	1.85	3.60	0.00005	0.00625
*CEACAM3*	0.63 (0.51–0.75)	1.36	2.56	0.00005	0.00625
*PANX2*	0.62 (0.49–0.74)	1.26	2.40	0.00005	0.00625
*LUCAT1*	0.61 (0.49–0.73)	1.21	2.31	0.00050	0.03779
*MANSC1*	0.61 (0.48–0.73)	1.13	2.19	0.00005	0.00625
*AOC2*	0.60 (0.48–0.73)	1.25	2.39	0.00005	0.00625
*LINC01002*	0.59 (0.47–0.72)	1.65	3.13	0.00005	0.00625
*MMP9*	0.58 (0.45–0.70)	1.80	3.48	0.00005	0.00625
*CXCR2*	0.58 (0.46–0.71)	1.57	2.96	0.00005	0.00625
*ADM*	0.58 (0.46–0.71)	1.28	2.43	0.00005	0.00625
*VNN3*	0.58 (0.45–0.70)	1.07	2.10	0.00005	0.00625
*KRT23*	0.57 (0.44–0.70)	1.59	3.01	0.00005	0.00625
*CXCL8*	0.57 (0.44–0.69)	1.32	2.50	0.00005	0.00625
*BMX*	0.57 (0.44–0.70)	1.30	2.45	0.00005	0.00625
*G0S2*	0.56 (0.44–0.69)	2.50	5.65	0.00155	0.08566
*CD177*	0.56 (0.43–0.69)	1.08	2.11	0.00025	0.023000
*CYP4F3*	0.55 (0.43–0.68)	1.42	2.68	0.00005	0.00625
*CHI3L1*	0.55 (0.43–0.68)	1.08	2.12	0.00005	0.00625
*ITGAD*	0.55 (0.42–0.68)	1.01	2.01	0.00005	0.00625
*ALPL*	0.54 (0.41–0.67)	2.83	7.09	0.00005	0.00625
*MME*	0.54 (0.41–0.67)	1.97	3.92	0.00005	0.00625
*CMTM2*	0.54 (0.41–0.67)	1.69	3.24	0.00005	0.00625
*MGAM*	0.54 (0.41–0.66)	1.11	2.16	0.00005	0.00625
*FCGR3B*	0.53 (0.40–0.66)	2.35	5.11	0.00005	0.00625
*AOC3*	0.53 (0.40–0.66)	1.58	2.99	0.00005	0.00625
*ADGRG3*	0.53 (0.40–0.66)	1.51	2.85	0.00005	0.00625
*HCAR3*	0.53 (0.41–0.66)	1.34	2.53	0.00005	0.00625

CI, confidence interval; AUC, area under the receiver operating characteristic curve.

**Table 3:** AUC for panels of DEGs that exhibited a log_2_(fold change) > 1.5 and AUC values > 0.6 and the combined *ADAMTS2* model with clinicopathological factors.

Models	AUC (95% Confidence Interval)
A	0.765 (0.658–0.872)
A + P + C + K	0.721 (0.609–0.833)
A + P + C	0.782 (0.682–0.881)
A + P	0.776 (0.674–0.879)
A + C	0.786 (0.687–0.886)
A + K	0.717 (0.605–0.830)
A + recipient age	0.799 (0.701–0.897)

A, *ADAMTS2*; P, *PLIN5*; C, *CLDN9*; K, *KCNJ15*; AUC, area under the receiver operating characteristic curve.

## Data Availability

Data are available upon reasonable request to the corresponding author.

## Supplementary Materials

The following supporting information can be downloaded at: https://www.mdpi.com/article/10.3390/jpm13081242/s1, Table S1: qPCR probe information; Table S2: Clinicopathological features for IF/TA patients and controls at one-year protocol biopsy after kidney transplantation; Table S3: qPCR validation of mRNA-sequencing data for IF/TA patients and controls at one-year protocol biopsy after kidney transplantation; Figure S1: (a) A density plot of FPKM distribution of all samples. A color code represents distinct samples. Each line in the density plot corresponds to distinct samples. FPKM, fragments of transcript per kilobase per million reads; I, IF/TA; C, Control (b) A box plot of FPKM distribution of all samples. The line in the box plot represents the median value, the box represents the interquartile range, and the whisker represents the highest and lowest values; Figure S2: ROC curves evaluating the sensitivity and specificity of the DEG, which showed a log_2_(fold change) of >1.5 and AUC values of >0.6 as a biomarker of IF/TA. AUC, area under the receiver operating characteristic curve; Figure S3: ROC curves evaluating the sensitivity and specificity of DEG panels with log_2_(fold change) >1.5 and AUC values >0.6 as biomarkers for IF/TA. The adjusted model represents a model according to *ADAMTS2* expression adjusted for recipient age. AUC, area under the receiver operating characteristic curve; A, *ADAMTS2*; P, *PLIN5*; C, *CLDN9*; K, *KCNJ15.*
