# Supplementary material for: Discovery of Biomarkers Related to Interstitial Fibrosis and Tubular Atrophy among Kidney Transplant Recipients by mRNA-Sequencing
Source: J Pers Med. 2023 Aug 10;13(8):1242. doi: 10.3390/jpm13081242 (PMC10455123; doi:10.3390/jpm13081242)
Supplement: Supplementary file 1 [file jpm-13-01242-s001.zip › jpm-2481578-supplementary.pdf]

Supplementary Material

Table S1. qPCR probe information.

	Gene ID	Assay ID
1	*ADAMTS2*	Hs01029111_m1
2	*CXCR2*	Hs01891184_s1
3	*IL1R2*	Hs00174759_m1
4	*MMP9*	Hs00957562_m1
5	*CHI3L1*	Hs01072228_m1
6	*LRG1*	Hs00364835_m1
7	*GAPDH*	Hs99999905_m1

Table S2. Clinicopathological features for IF/TA patients and controls at one-year protocol biopsy after kidney transplantation

Sample	Banff scores	Diagnosis	Recipient age	Sex	BMI	Dialysis	Original disease	Donor type	Donor age	POD	eGFR biopsy	eGFR sampling
I1	t1, v0, i2, g0, ti2, i-IFTA1 / ci1, ct2, cg0, mm0, cv0, ah0, aah0 / ptc0	IF/TA grade 2, Borderline TCMR	47	M	25.29	ND	DM	LU	45	780	62.8	64.4
I2	t1, v0, i1, g0, ti1, i-IFTA1 / ci1, ct1, cg0, mm0, cv0, ah0, aah0 / ptc0	IF/TA grade 1, Borderline TCMR	27	M	20.74	HD	GN	LR	53	610	64.3	84.4
I3	t1, v0, i0, g0, ti0, i-IFTA1 / ci0, ct1, cg0, mm0, cv0, ah0, aah0 / ptc0	IF/TA grade 1, Borderline TCMR	53	F	17.80	HD	GN	D	66	986	53.9	63.9
I4	t0, v0, i1, g0 / ci0, ct1, cg0, mm0, cv0, ah0 / ptc0	IF/TA grade 1	44	F	19.97	HD	GN	D	49	1259	59.1	73.7
I5	t1, v0, i1, g0 / ci1, ct1, cg0, mm0, cv2, ah0 / ptc0	IF/TA grade 1, Borderline TCMR	49	M	22.60	HD	others	D	45	1637	70.1	68.6
I6	t2, v0, i1, g0, ti1, i-IFTA2 / ci1, ct1, cg0, mm0, cv2, ah0, aah0 / ptc0	IF/TA grade 1, Borderline TCMR	50	M	23.72	HD	UK	LR	52	806	55.7	51.9
I7	t1, v0, i1, g0, ti1, i-IFTA1 / ci0, ct1, cg0, mm0, cv2, ah0, aah0 / ptc0	IF/TA grade 1, Borderline TCMR	33	M	25.91	ND	others	LR	65	638	48.3	50.5
I8	t0, v0, i1, g0, ti1, i-IFTA1 / ci1, ct0, cg0, mm0, cv1, ah0, aah0 / ptc0	IF/TA grade 1	67	M	20.44	HD	UK	D	46	814	59.0	66.4
I9	t0, v0, i0, g0, ti0, i-IFTA0 / ci0, ct1, cg0, mm0, cv0, ah0, aah0 / ptc0	IF/TA grade 1	64	F	29.12	ND	UK	LR	42	593	80.0	73.0
I10	t1, v0, i0, g0, ti1, i-IFTA2 / ci2, ct2, cg0, mm0, cv2, ah0, aah0 / ptc0	IF/TA grade 1, Borderline TCMR	61	F	21.29	HD	UK	D	71	745	40.8	42.5
I11	t1, v0, i2, g1, ti2, i-IFTA1 / ci1, ct1, cg0, mm0, cv0, ah0, aah0 / ptc0	IF/TA grade 1, Borderline TCMR	55	M	22.30	HD	DM	LU	50	636	59.2	56.9
I12	t1, v0, i1, g0, ti2, i-IFTA1 / ci1, ct2, cg0, mm0, cv1, ah0, aah0 / ptc1	IF/TA grade 2, Borderline TCMR	34	F	18.18	PD	PKD	D	34	1174	50.5	48.2
I13	t2, v0, i1, g0 / ci2, ct2, cg0, mm0, cv2, ah0 / ptc0	IF/TA grade 2, Borderline TCMR	61	M	23.59	ND	others	LR	48	1279	75.4	84.0
I14	t1, v0, i1, g0 / ci1, ct1, cg0, mm0, cv0, ah0 / ptc0	IF/TA grade 1, Borderline TCMR	64	F	20.67	HD	GN	LR	55	1056	79.8	84.9
I15	t1, v0, i0, g0, ti0, i-IFTA0 / ci0, ct1, cg0, mm0, cv0, ah0, aah0 / ptc0	IF/TA grade 1, Borderline TCMR	44	M	27.80	HD	GN	LU	41	477	40.7	50.6
I16	t0, v0, i0, g0, ti0, i-IFTA0 / ci0, ct1, cg0, mm0, cv0, ah0, aah0 / ptc0	IF/TA grade 1	56	M	24.45	PD	DM	LU	52	684	62.4	73.4
I17	t0, v0, i0, g0, ti0, i-IFTA1 / ci0, ct1, cg0, mm0, cv0, ah0, aah0 / ptc0	IF/TA grade 1	36	M	30.07	HD	UK	LR	62	1035	52.9	56.4
I18	t1, v0, i1, g0, ti1, i-IFTA1 / ci0, ct1, cg0, mm0, cv1, ah0, aah0 / ptc0	IF/TA grade 1, Borderline TCMR	51	M	24.35	HD	DM	LU	43	708	66.5	51.7

I19	t1, v0, i1, g0, ti1, i-IFTA0 / ci1, ct1, cg0, mm0, cv1, ah0, aah0 / ptc0	IF/TA grade 1, Borderline TCMR	58	F	20.04	HD	UK	D	NA	789	40.8	39.3
I20	t1, v0, i0, g0, ti1, i-IFTA1 / ci1, ct1, cg0, mm0, cv0, ah0, aah0 / ptc0	IF/TA grade 1, Borderline TCMR	39	F	31.79	ND	PKD	LR	41	435	60.0	57.8
I21	t0, v0, i0, g0, ti0, i-IFTA0 / ci0, ct1, cg0, mm0, cv2, ah0, aah0 / ptc0	IF/TA grade 1	65	M	24.55	HD	PKD	D	69	460	63.6	71.5
I22	t0, v0, i0, g0, ti1, i-IFTA1 / ci0, ct1, cg0, mm0, cv2, ah0, aah0 / ptc0	IF/TA grade 1	60	M	23.05	ND	GN	LR	57	887	58.7	65.1
I23	t1, v0, i1, g0, ti1, i-IFTA2 / ci0, ct1, cg0, mm0, cv0, ah0, aah0 / ptc0	IF/TA grade 1, Borderline TCMR	58	F	22.96	HD	DM	D	34	911	68.3	54.7
I24	t1, v0, i1, g0, ti2, i-IFTA2 / ci1, ct1, cg0, mm0, cv1, ah0, aah0 / ptc1	IF/TA grade 1, Borderline TCMR	43	F	35.32	HD	DM	D	35	576	68.0	59.3
I25	t0, v0, i1, g0 / ci1, ct1, cg0, mm0, cv0, ah0 / ptc0	IF/TA grade 1	36	M	23.82	HD	HTN	LR	33	1833	62.9	57.7
I26	t0, v0, i0, g0, ti0, i-IFTA0 / ci1, ct1, cg0, mm0, cv1, ah0, aah0 / ptc0	IF/TA grade 1	58	M	27.52	HD	DM	D	30	1271	59.5	68.8
I27	t0, v0, i0, g0, ti0, i-IFTA0 / ci1, ct1, cg0, mm0, cv1, ah1, aah0 / ptc0	IF/TA grade 1	41	F	18.14	ND	GN	LR	40	625	53.3	52.0
I28	t1, v0, i1, g1, ti1, i-IFTA2 / ci1, ct1, cg0, mm0, cv0, ah0, aah0 / ptc0	IF/TA grade 1, Borderline TCMR	52	F	25.37	HD	GN	D	50	783	63.1	78.1
I29	t0, v0, i0, g0, ti1, i-IFTA1 / ci0, ct1, cg0, mm0, cv0, ah0, aah0 / ptc0	IF/TA grade 1	34	M	16.22	HD	GN	D	NA	953	79.4	70.1
I30	t0, v0, i1, g0 / ci1, ct1, cg0, mm0, cv1, ah0 / ptc0	IF/TA grade 1	50	F	22.89	PD	GN	D	74	1686	35.4	26.2
I31	t0, v0, i0, g0 / ci0, ct0, cg0, mm0, cv0, ah0 / ptc0	IF/TA grade 1, Borderline TCMR	56	F	21.71	HD	HTN	D	NA	1411	57.6	59.3
I32	t1, v0, i2, g0, ti2, i-IFTA2 / ci1, ct1, cg0, mm0, cv1, ah0, aah0 / ptc2	IF/TA grade 1, Borderline TCMR	50	F	19.49	HD	PKD	LR	54	1091	65.1	66.3
I33	t1, v0, i1, g0 / ci0, ct1, cg0, mm0, cv0, ah0 / ptc0	IF/TA grade 1, Borderline TCMR	40	F	18.18	HD	GN	D	37	1508	78.9	75.7
I34	t1, v0, i0, g0, ti0, i-IFTA0 / ci0, ct1, cg0, mm0, cv0, ah0, aah0 / ptc0	IF/TA grade 1, Borderline TCMR	46	M	23.36	HD	DM	D	25	636	103.6	97.5
I35	t1, v0, i1, g0, ti1, i-IFTA2 / ci0, ct1, cg0, mm0, cv0, ah0, aah0 / ptc0	IF/TA grade 1, Borderline TCMR	64	M	26.98	PD	UK	LU	61	531	70.9	56.4
I36	t0, v0, i0, g0, ti0, i-IFTA0 / ci0, ct1, cg0, mm0, cv1, ah0, aah0 / ptc0	IF/TA grade 1	46	F	26.22	ND	PKD	LU	46	642	64.6	59.2
I37	t1, v0, i1, g0 / ci1, ct1, cg0, mm0, cv0, ah0 / ptc0	IF/TA grade 1, Borderline TCMR	41	F	21.15	HD	UK	LR	62	1786	47.7	47.0
I38	t1, v0, i0, g0, ti1, i-IFTA1 / ci1, ct1, cg1a, mm0, cv1, ah0, aah0 / ptc0	IF/TA grade 1, Borderline TCMR	65	M	23.49	HD	PKD	D	70	630	55.2	58.7

I39	t1, v0, i1, g1 / ci1, ct1, cg0, mm0, cv1, ah0 / ptc0	IF/TA grade 1, Borderline TCMR	58	F	20.40	HD	GN	LR	43	1853	48.2	60.1
I40	t1, v0, i1, g0 / ci1, ct1, cg0, mm0, cv1, ah0 / ptc0	IF/TA grade 1, Borderline TCMR	30	F	19.20	PD	GN	LR	56	1951	84.6	66.9
I41	t1, v0, i0, g0, ti1, i-IFTA2 / ci2, ct2, cg0, mm0, cv0, ah0, aah0 / ptc0	IF/TA grade 2, Borderline TCMR	56	M	23.50	ND	PKD	LU	57	967	64.7	67.1
C1	t0, v0, i0, g0, ti0, i-IFTA0 / ci0, ct0, cg0, mm0, cv0, ah0, aah0 / ptc0	no evidence of acute rejection	58	M	25.14	HD	DM	LR	20	819	80.1	94.5
C2	t0, v0, i0, g0 / ci0, ct0, cg0, mm0, cv0, ah0 / ptc0	no evidence of acute rejection	35	M	22.05	HD	GN	D	42	1780	69.8	60.9
C3	t0, v0, i0, g0 / ci0, ct0, cg0, mm1, cv1, ah0 / ptc0	no evidence of acute rejection	56	F	21.05	HD	others	LU	57	1133	77.0	74.5
C4	t0, v0, i0, g0, ti0, i-IFTA0 / ci0, ct0, cg0, mm0, cv1, ah0, aah0 / ptc0	no evidence of acute rejection	68	M	23.31	HD	PKD	LU	68	1070	53.2	68.1
C5	t0, v0, i0, g0, ti0, i-IFTA0 / ci0, ct0, cg0, mm0, cv1, ah0, aah0 / ptc0	no evidence of acute rejection	43	M	25.69	HD	HTN	LU	41	839	73.1	74.4
C6	t0, v0, i0, g0 / ci0, ct0, cg0, mm0, cv1, ah0 / ptc0	no evidence of acute rejection	61	M	20.24	HD	GN	D	65	1103	55.4	64.0
C7	t0, v0, i0, g0, ti0, i-IFTA0 / ci0, ct0, cg0, mm0, cv0, ah0, aah0 / ptc0	no evidence of acute rejection	21	F	17.85	ND	others	LR	47	955	78.9	69.7
C8	t0, v0, i0, g0, ti0, i-IFTA0 / ci0, ct0, cg0, mm0, cv0, ah0, aah0 / ptc0	no evidence of acute rejection	31	F	30.14	HD	UK	LR	53	798	50.8	42.7
C9	t0, v0, i0, g0 / ci0, ct0, cg0, mm0, cv0, ah0 / ptc0	no evidence of acute rejection	37	M	24.32	HD	GN	LR	40	1195	72.6	71.1
C10	t0, v0, i0, g0, ti0, i-IFTA0 / ci0, ct0, cg0, mm0, cv1, ah0, aah0 / ptc0	no evidence of acute rejection	68	M	26.29	ND	UK	LU	63	586	50.9	55.1
C11	t0, v0, i0, g0, ti0, i-IFTA0 / ci0, ct0, cg0, mm0, cv1, ah0, aah0 / ptc0	no evidence of acute rejection	51	M	23.92	HD	UK	LR	54	839	66.6	71.5
C12	t0, v0, i0, g0 / ci0, ct0, cg0, mm0, cv0, ah0 / ptc0	no evidence of acute rejection	58	M	27.91	HD	DM	D	27	1057	97.4	75.1
C13	t0, v0, i0, g0, ti0, i-IFTA0 / ci0, ct0, cg0, mm0, cv0, ah0, aah0 / ptc0	no evidence of acute rejection	63	F	20.66	HD	GN	LU	67	996	80.2	75.0
C14	t0, v0, i0, g0 / ci0, ct0, cg0, mm0, cv1, ah0 / ptc0	no evidence of acute rejection	20	M	20.64	PD	others	LR	51	2008	64.4	49.0
C15	t0, v0, i0, g0, ti0, i-IFTA0 / ci0, ct0, cg0, mm0, cv1, ah0, aah0 / ptc0	no evidence of acute rejection	64	M	19.93	HD	GN	LR	39	687	70.1	69.1
C16	t0, v0, i0, g0 / ci0, ct0, cg0, mm1, cv1, ah0 / ptc0	no evidence of acute rejection	47	F	21.01	HD	DM	LR	65	1122	77.6	77.0
C17	t0, v0, i0, g0 / ci1, ct0, cg0, mm0, cv1, ah0 / ptc0	no evidence of acute rejection	20	F	17.53	ND	GN	LR	49	1319	61.7	61.0

C18	t0, v0, i0, g0 / ci0, ct0, cg0, mm0, cv0, ah0 / ptc0	no evidence of acute rejection	63	F	23.04	HD	GN	LR	46	1160	60.6	65.1
C19	t0, v0, i0, g0, ti0, i-IFTA0 / ci0, ct0, cg0, mm0, cv0, ah0, aah0 / ptc0	no evidence of acute rejection	63	M	25.19	HD	DM	LR	37	832	56.6	56.4
C20	t0, v0, i0, g0, ti0, i-IFTA0 / ci0, ct0, cg0, mm0, cv0, ah0, aah0 / ptc0	no evidence of acute rejection	57	F	27.52	HD	UK	D	33	508	51.0	48.5
C21	t0, v0, i0, g0, ti0, i-IFTA0 / ci0, ct0, cg0, mm0, cv0, ah0, aah0 / ptc0	no evidence of acute rejection	33	M	24.21	PD	GN	LR	60	599	54.8	49.1
C22	t0, v0, i1, g0 / ci0, ct0, cg0, mm0, cv0, ah0 / ptc0	no evidence of acute rejection	60	M	24.91	HD	DM	LU	58	1305	64.0	57.8
C23	t0, v0, i0, g0 / ci0, ct0, cg0, mm1, cv0, ah0 / ptc0	no evidence of acute rejection	29	M	30.07	ND	GN	LR	48	1140	53.2	61.8
C24	t0, v0, i0, g0 / ci0, ct0, cg0, mm0, cv0, ah0 / ptc0	no evidence of acute rejection	52	F	23.91	HD	others	LR	30	1133	57.3	71.4
C25	t0, v0, i0, g0 / ci0, ct0, cg0, mm0, cv0, ah0 / ptc0	no evidence of acute rejection	45	F	20.56	ND	UK	LU	48	1534	63.9	62.5
C26	t0, v0, i0, g0, ti0, i-IFTA0 / ci0, ct0, cg0, mm0, cv0, ah0, aah0 / ptc0	no evidence of acute rejection	52	F	24.50	ND	DM	LR	29	694	68.1	66.1
C27	t0, v0, i0, g0, ti0, i-IFTA0 / ci0, ct0, cg0, mm0, cv0, ah0, aah0 / ptc0	no evidence of acute rejection	39	F	26.50	HD	DM	D	24	1042	80.3	81.0
C28	t0, v0, i0, g0, ti0, i-IFTA0 / ci0, ct0, cg0, mm0, cv1, ah0, aah0 / ptc0	no evidence of acute rejection	62	M	26.84	ND	DM	LR	27	538	54.8	55.8
C29	t0, v0, i0, g0 / ci0, ct0, cg0, mm0, cv0, ah0 / ptc0	no evidence of acute rejection	61	M	20.47	HD	PKD	D	58	1880	54.8	56.6
C30	t0, v0, i0, g0 / ci0, ct0, cg0, mm0, cv0, ah0 / ptc0	no evidence of acute rejection	44	F	20.91	HD	GN	LR	35	1329	75.4	80.4
C31	t0, v0, i0, g0 / ci0, ct0, cg0, mm0, cv0, ah0 / ptc0	no evidence of acute rejection	57	M	22.68	HD	GN	D	23	1290	70.8	75.1
C32	t0, v0, i1, g0 / ci0, ct0, cg0, mm0, cv0, ah0 / ptc0	no evidence of acute rejection	34	F	22.59	ND	DM	LR	58	1828	62.8	62.4
C33	t0, v0, i0, g0 / ci0, ct0, cg0, mm0, cv0, ah0 / ptc0	no evidence of acute rejection	42	M	26.19	ND	others	LR	43	1553	61.2	67.0
C34	t0, v0, i1, g0, ti1, i-IFTA0 / ci0, ct0, cg0, mm0, cv0, ah0, aah0 / ptc0	no evidence of acute rejection	57	F	18.83	HD	PKD	LU	57	528	69.6	62.5
C35	t0, v0, i0, g0, ti0, i-IFTA0 / ci0, ct0, cg0, mm0, cv0, ah0, aah0 / ptc0	no evidence of acute rejection	55	F	20.06	HD	GN	LU	59	519	74.2	81.2
C36	t0, v0, i0, g0 / ci0, ct0, cg0, mm0, cv0, ah0 / ptc0	no evidence of acute rejection	63	M	19.10	HD	GN	LU	60	1235	46.7	51.6
C37	t0, v0, i0, g0 / ci0, ct0, cg0, mm1, cv0, ah0 / ptc0	no evidence of acute rejection	36	M	22.59	ND	GN	LU	34	1501	82.7	77.8

C38	t0, v0, i1, g0 / ci0, ct0, cg0, mm0, cv0, ah0 / ptc0	no evidence of acute rejection	50	M	24.20	HD	UK	D	53	1894	52.9	55.8
C39	t0, v0, i0, g0 / ci0, ct0, cg0, mm0, cv0, ah0 / ptc1	no evidence of acute rejection	68	M	23.39	HD	HTN	LR	39	1500	79.5	59.5
C40	t0, v0, i0, g0, ti0, i-IFTA0 / ci0, ct0, cg0, mm0, cv0, ah0, aah0 / ptc0	no evidence of acute rejection	55	M	27.40	HD	UK	LR	41	624	47.7	57.6
C41	t0, v0, i0, g0, ti0, i-IFTA0 / ci0, ct0, cg0, mm0, cv0, ah0, aah0 / ptc0	no evidence of acute rejection	39	M	26.93	HD	others	LR	42	559	70.4	60.1

I, IF/TA; C, Control; IT/FA, interstitial fibrosis and tubular atrophy; M, male; F, female; BMI, body mass index; ND, no dialysis; HD, hemodialysis; PD, peritoneal dialysis; DM, diabetes; GN, glomerular nephritis; HTN, hypertension; PKD, polycystic kidney disease; UK, unknown; D, deceased; LR, living/related; LU, living/unrelated; POD, postoperative days; eGFR, estimated glomerular filtration rate; NA, not available; TCMR, T cell-mediated rejection; t0, cortical tubules without tubulitis; t1, defined as foci of 1-4 mononuclear cells per tubular cross section or per 10 tubular epithelial cells; t2, defined as 5 to 10 mononuclear cells per tubular cross section or per 10 epithelial cells; t3, defined as foci with >10 mononuclear cells/tubular cross section; v0, no arteritis; v1, mild to moderate intimal arteritis in at least 1 arterial cross section; v2, severe intimal arteritis with at least 25% luminal area lost in at least 1 arterial cross section; v3, transmural arteritis and/or arterial fibrinoid change and medial smooth muscle necrosis with lymphocytic infiltrate in vessel; i0, no inflammation or in less than 10% of unscarred cortical parenchyma; i1, inflammation in 10 to 25% of unscarred cortical parenchyma; i2, inflammation in 26 to 50% of unscarred cortical parenchyma; i3, inflammation in more than 50% of unscarred cortical parenchyma; g0, no glomerulitis; g1, segmental or global glomerulitis in less than 25% of glomeruli; g2, segmental or global glomerulitis in 25 to 75% of glomeruli; g3, segmental or global glomerulitis in more than 75% of glomeruli; ti0, no or trivial interstitial inflammation (<10% of total cortical parenchyma); ti1, 10-25% of total cortical parenchyma inflamed; ti2, 26-50% of total cortical parenchyma inflamed; ti3, >50% of total cortical parenchyma inflamed; ptc0, maximum number of leukocytes <3; ptc1, at least 1 leukocyte cell in ≥10% of cortical PTCs with 3-4 leukocytes in most severely involved PTC; ptc2, at least 1 leukocyte in ≥10% of cortical PTC with 5-10 leukocytes in most severely involved PTC; ptc3, at least 1 leukocyte in ≥10% of cortical PTC with >10 leukocytes in most severely involved PTC; ci0, interstitial fibrosis in up to 5% of cortical area; ci1, interstitial fibrosis in 6 to 25% of cortical area (mild interstitial fibrosis); ci2, interstitial fibrosis in 26 to 50% of cortical area (moderate interstitial fibrosis); ci3, interstitial fibrosis in >50% of cortical area (severe interstitial fibrosis); ct0, no tubular atrophy; ct1, tubular atrophy involving up to 25% of the area of cortical tubules; ct2, tubular atrophy involving 26 to 50% of the area of cortical tubules; ct3, tubular atrophy involving in >50% of the area of cortical tubules; cv0, no chronic vascular changes; cv1, vascular narrowing of up to 25% luminal area by fibrointimal thickening; cv2, vascular narrowing of 26 to 50% luminal area by fibrointimal thickening; cv3, vascular narrowing of more than 50% luminal area by fibrointimal thickening; cg0, no glomerular basement membrane (GBM) double contours by light microscopy (LM) or electron microscopy (EM); cg1a, no GBM double contours by LM but GBM double contours (incomplete or circumferential) in at least 3 glomerular capillaries by EM, with associated endothelial swelling and/or subendothelial electron-lucent widening; cg1b, double contours of the GBM in 1-25% of capillary loops in the most affected nonsclerotic glomerulus by LM; EM confirmation is recommended if EM is available; cg2, double contours affecting 26 to 50% of peripheral capillary loops in the most affected, glomerulus; cg3, double contours affecting more than 50% of peripheral capillary loops in the most affected-glomerulus; mm0, no more than mild mesangial matrix increase in any glomerulus; mm1, at least moderate mesangial matrix increase in up to 25% of nonsclerotic glomeruli; mm2, at least moderate mesangial matrix increase in 26% to 50% of nonsclerotic glomeruli; mm3, at least moderate mesangial matrix increase in >50% of nonsclerotic glomeruli; ah0, no periodic acid-Schiff (PAS)-positive hyaline arteriolar thickening; ah1, mild to moderate PAS-positive hyaline thickening in at least 1 arteriole; ah2, moderate to severe PAS-positive hyaline thickening in more than 1 arteriole; ah3, Severe PAS-positive hyaline thickening in many arterioles; aah0, no typical lesions of calcineurin inhibitor-related arteriolopathy; aah1, replacement of degenerated smooth muscle cells by hyaline deposits in only 1 arteriole, without circumferential involvement; aah2, replacement of degenerated smooth muscle cells by hyaline deposits in more than 1 arteriole, without circumferential involvement; aah3, replacement of degenerated smooth muscle cells by hyaline deposits with circumferential involvement, independent of the number of arterioles involved; i-IFTA0, no inflammation or less than 10% of scarred cortical

parenchyma; i-IFTA1, inflammation in 10% to 25% of scarred cortical parenchyma; i-IFTA2, inflammation in 26% to 50% of scarred cortical parenchyma; i-IFTA3, inflammation in >50% of scarred cortical parenchyma[1]

[1] Roufosse C, Simmonds N, Clahsen-van Groningen M, Haas M, Henriksen KJ, Horsfield C, et al. A 2018 Reference Guide to the Banff Classification of Renal Allograft Pathology. Transplantation 2018; 102: 1795–1814

Table S3. qPCR validation of mRNA-sequencing data for IF/TA patients and controls at one-year protocol biopsy after kidney transplantation.

Gene ID	mRNA sequencing		qPCR	
	Fold change[a]	*q*	Fold change[a]	*p*
ADAMTS2	3.52	0.006	2.20	<0.001
CHI3L1	2.12	0.006	1.53	0.012
CXCR2	2.96	0.006	1.39	0.002
IL1R2	2.68	0.006	1.75	<0.001
LRG1	2.19	0.006	1.55	<0.001
MMP9	3.48	0.006	1.72	0.001

[a] Fold change in the mean value (IF/TA vs. control).

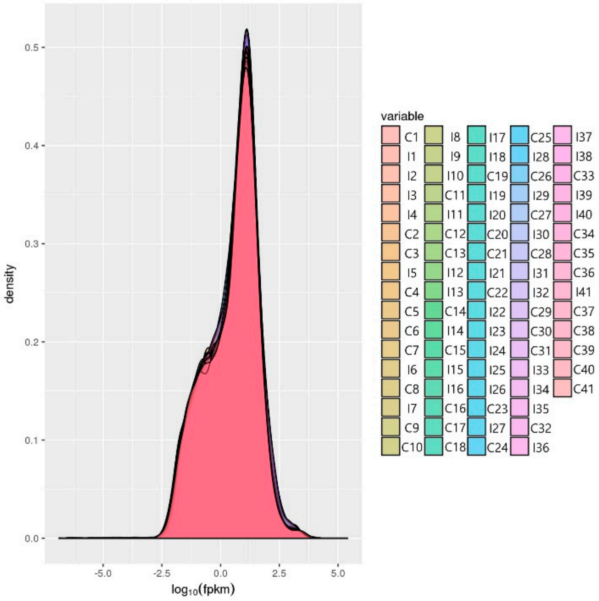

(a)

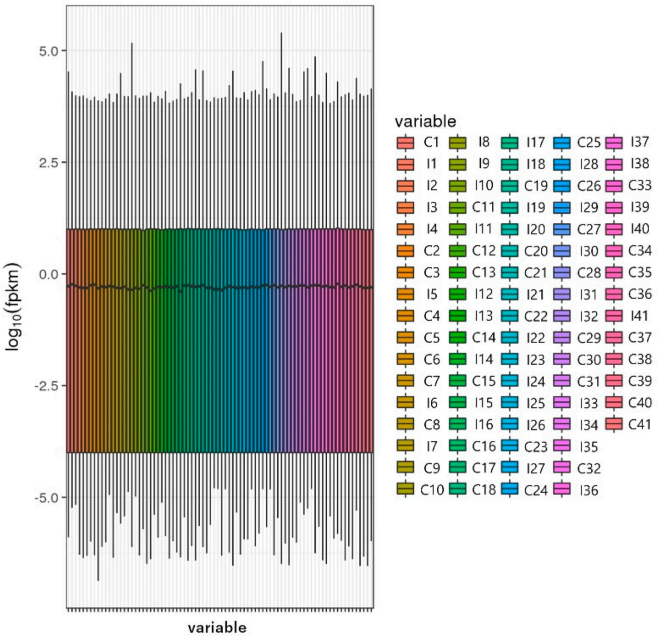

(b)

Figure S1. (**a**) A density plot of FPKM distribution of all samples. A color code represents distinct samples. Each line in the density plot corresponds to distinct samples. FPKM, fragments of transcript per kilobase per million reads; I, IF/TA; C, Control (**b**) A box plot of FPKM distribution of all samples. The line in the box plot represents the median value, the box represents the interquartile range, and the whisker represents the highest and lowest values.

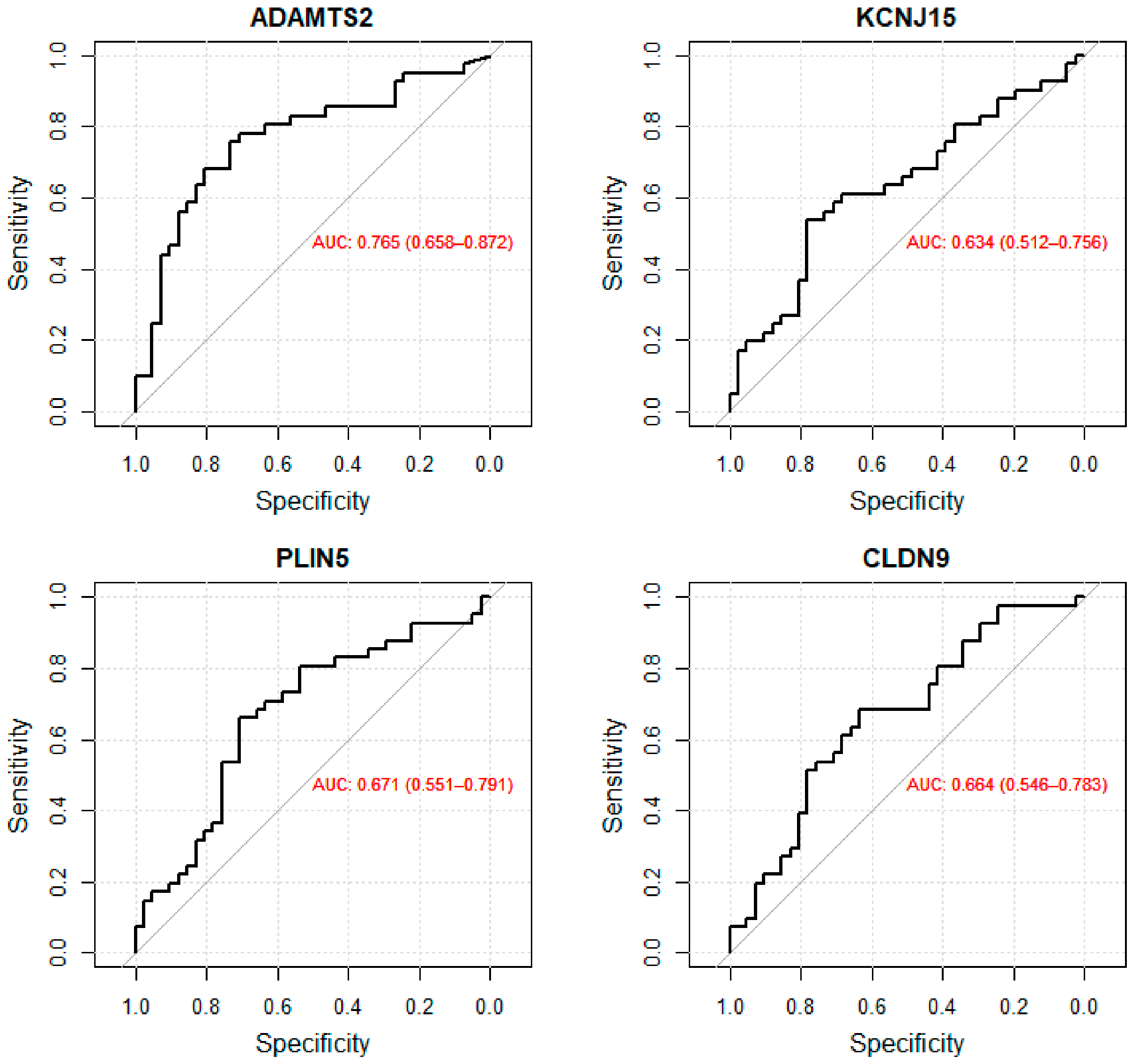

Figure S2. ROC curves evaluating the sensitivity and specificity of the DEG's which showed a $\log_2$(fold change) of > 1.5 and AUC values of > 0.6 as a biomarker of IF/TA. AUC, area under the receiver operating characteristic curve

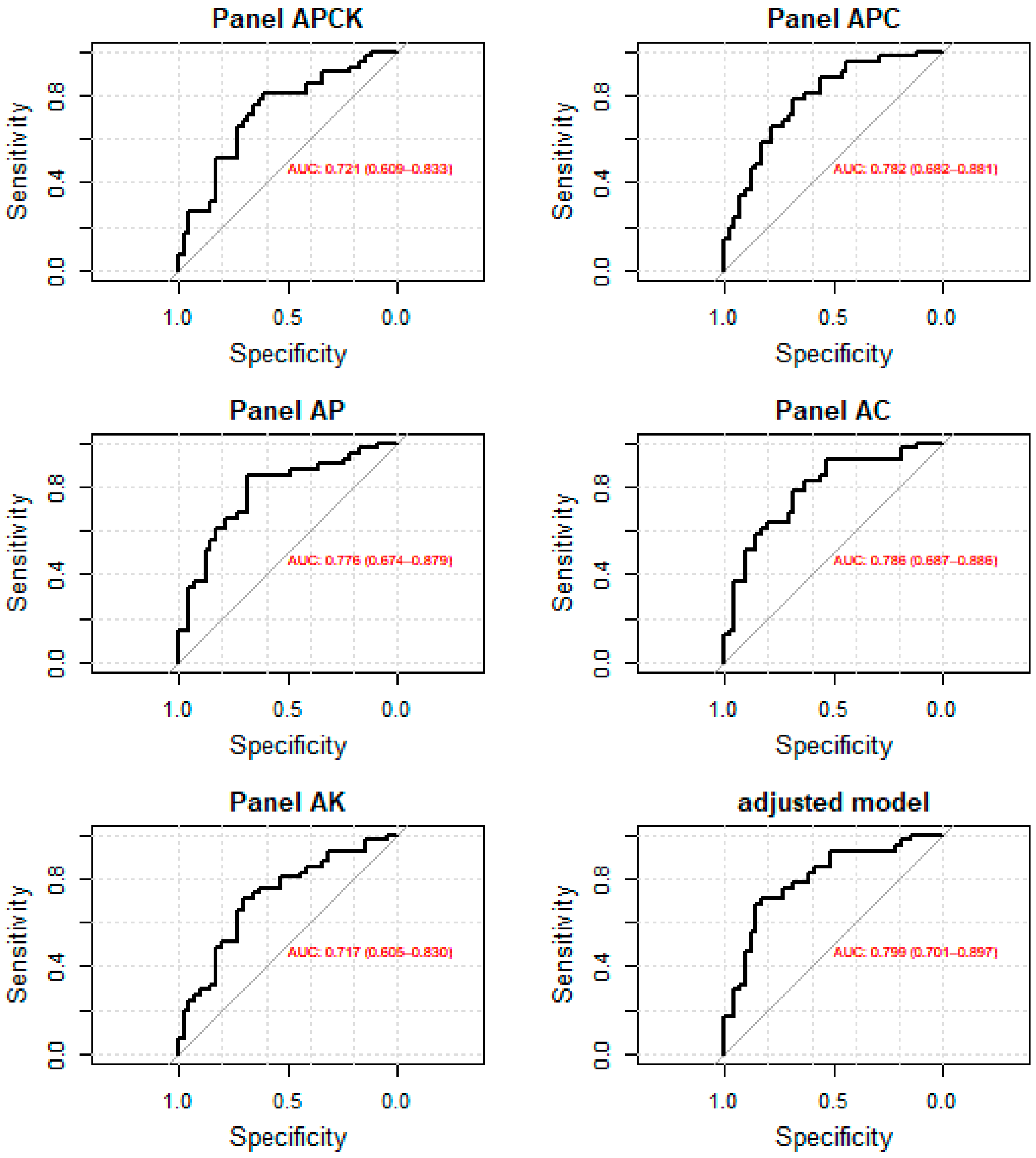

Figure S3. ROC curves evaluating the sensitivity and specificity of DEG panels with $\log_2$ (fold change) >1.5 and AUC values >0.6 as biomarkers for IF/TA. The adjusted model represents a model according to *ADAMTS2* expression adjusted for recipient age. AUC, area under the receiver operating characteristic curve; A, *ADAMTS2*; P, *PLIN5*; C, *CLDN9*; K, *KCNJ15*
